# The influence of alpha-melanocortin enantiomers on acetaminophen-induced hepatis in mice

**DOI:** 10.1186/2050-6511-13-S1-A77

**Published:** 2012-09-17

**Authors:** Petra Turčić, Karlo Houra, Mirna Bradamante, Paško Konjevoda, Tomislav Kelava, Nikola Štambuk

**Affiliations:** 1 Department of Pharmacology, Faculty of Pharmacy and Biochemistry, University of Zagreb, 10000 Zagreb, Croatia; 2 St. Catherine’s Hospital, 49210 Zabok, Croatia; 3 Department of Dermatology and Venerology, University Hospital Center Zagreb, 10000 Zagreb, Croatia; 4 Ruđer Bošković Institute, 10000 Zagreb, Croatia; 5 Department of Physiology and Immunology, School of Medicine, University of Zagreb, 10000 Zagreb, Croatia

## Background

L-alpha-Melanocortin is a strong inhibitor of inflammation. It is a promising new anti-inflammatory and hepatoprotective peptide. Consequently, its melanocortin receptors (MC_1_, MC_3_, MC_4_ and MC_5_) could be possible targets for the development of new antiinflammatory drugs for chronic inflammatory liver disease. For a long time it has been believed that only the L-enantiomers of amino acids are present in higher animals, but recent investigations show that D-amino acids also exhibit physiological effects *in vivo*, despite their very small quantities. The aim of this study was to compare hepatoprotective effects of L-alpha-melanocortin and D-alpha-melanocortin using the acetaminophen model of chemical liver damage in male CBA mice.

## Methods

Tested substances were applied intraperitoneally 60 minutes prior to the intragastric application of acetaminophen (150 mg/kg). Animals were sacrificed 24 hours after the administration of acetaminophen. The criteria for monitoring hepatoprotective effects of the tested substances were biochemical parameters (AST and ALT) and histopathological analysis.

## Results

The results obtained by the histopathological analysis and biochemical findings show potent hepatoprotective and anti-inflammatory effects of L-alpha-melanocortin in the liver, and suggest the possibility of modulating liver inflammation by means of melanocortin molecules and related receptors. D-alpha-melanocortin did not show any hepatoprotective effects *in vivo*.

## Conclusions

Our results show that peptide enantiomerism influences the protective effects of alpha-melanocortin peptides *in vivo*. This concept may be used to modulate peptide function *in vivo* and antibody binding assay *in vitro*.

